# Beraprost Attenuates Starvation‐Induced Apoptosis and Protects Feline Endothelial Cells

**DOI:** 10.1002/vms3.71127

**Published:** 2026-08-10

**Authors:** Takumi Matsuura, Cris Niño Bon B. Marasigan, Tomohiro Yonezawa

**Affiliations:** ^1^ Toray Industries Inc. Tokyo Japan; ^2^ Laboratory of Veterinary Clinical Pathology Department of Veterinary Medical Sciences Graduate School of Agricultural and Life Sciences The University of Tokyo Tokyo Japan; ^3^ Department of Veterinary Paraclinical Sciences College of Veterinary Medicine, University of the Philippines Los Baños Laguna Philippines

**Keywords:** beraprost, cats, chronic kidney disease, endothelial cells, feline, starvation‐induced apoptosis

## Abstract

**Objectives:**

This study aimed to establish an in vitro model of feline endothelial cells (FECs) undergoing apoptosis when cultured under various nutrient deprivation (starvation) conditions to investigate the protective effects of beraprost and its stereoisomers on endothelial cells.

**Methods:**

Various starvation conditions were tested to induce apoptosis in immortalized feline aortic endothelial cells as assessed by measuring caspase‐3/7 activity. The anti‐apoptotic effects of the racemic mixture of beraprost and the stereoisomers beraprost‐314d and beraprost‐315d contained in the racemic mixture were determined by measuring caspase‐3/7 activity and *Bax*/*Bcl2* gene expression and performing the terminal deoxynucleotidyl transferase dUTP nick end labeling (TUNEL) assay.

**Results:**

Starvation significantly increased caspase‐3/7 activity and induced apoptosis in FECs. Beraprost and beraprost‐314d efficiently inhibited caspase‐3/7 activation in a dose‐dependent manner, whereas beraprost‐315d exhibited lower potency. Beraprost downregulated *Bax* expression and reduced the number of TUNEL‐positive cells, confirming its anti‐apoptotic effects.

**Conclusion and Relevance:**

Beraprost effectively attenuates starvation‐induced apoptosis‐associated events in cultured immortalized feline aortic endothelial cells, inhibits caspase‐3/7 activity, and modulates *Bax* expression. These findings suggest a potential hypothesized mechanism that may contribute to the clinical efficacy of beraprost observed in feline CKD, although further validation using renal endothelial cells and in vivo models is required.

## Graphical abstract

This study established a feline endothelial cell starvation model to test the anti‐apoptotic effects of beraprost and its stereoisomers. Beraprost and beraprost‐314d reduced starvation‐induced apoptosis by inhibiting caspase‐3/7 activity and modulating Bax expression, while beraprost‐315d was less potent. These findings suggest a potential mechanism for beraprost's efficacy in cats.

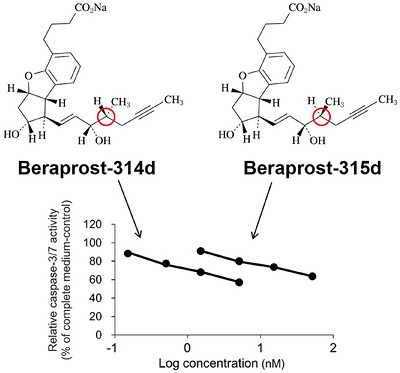

## 1 Introduction

Vascular endothelial cell senescence and apoptosis and the resulting capillary rarefaction, characterized by reduced density of microvascular networks, are hallmarks of age‐related kidney disorders (Tesfamariam and DeFelice 2007; Jourde‐Chiche et al. 2019; Li et al. 2022). In feline patients with chronic kidney disease (CKD), evidence of capillary rarefaction using quantitative immunohistochemistry in the affected kidneys shows a positive correlation with kidney dysfunction among other histopathological lesions (Paschall et al. 2023). Clinical data suggest that slowing the loss of kidney function by preserving the renal endothelium and capillary network functions (Goto et al. 2014) may prolong the lifespan of animals with CKD (Yamaguchi et al. 2013).

Beraprost is a chemically stable synthetic analogue of prostacyclin with high and long‐lasting activity after oral administration (Toda 1988; Niwano et al. 2003; Matsumoto et al. 2002). A previous clinical study suggested an association between beraprost treatment and changes in serum creatinine levels in feline patients with CKD (Takenaka et al. 2018); however, the direct effects of beraprost on overall survival and specific renal endothelial protection remain to be fully elucidated. Recently, Ito et al. (2023) reported that beraprost therapy is significantly associated with improved overall survival in cats with CKD. Beraprost is a racemic mixture consisting of two pairs of stereoisomers, beraprost‐314d and beraprost‐315d, and their enantiomers, in a total molar ratio of 1:1:1:1. Published data provide evidence that different isomers of beraprost possess different pharmacological potencies (Kajikawa et al. 1989; Shen et al. 2019); however, it is unknown whether these effects are species‐ and/or cell type‐specific, and what kind of effects they would induce in feline endothelial cells (FECs).

For in vitro experiments with FECs, the previously described immortalized FEC line was established using the simian virus 40 large T antigen (SV40LT) and the catalytic subunit of human telomerase reverse transcriptase (hTERT) (Olyslaegers et al. 2013). FECs have been shown to largely maintain the structural and functional phenotypes of primary endothelial cells, as well as an immunologic response to tumour necrosis factor (Olyslaegers et al. 2013), an inducer of apoptosis.

This study aimed to establish an in vitro FEC model using various nutritional starvation conditions to induce apoptosis and investigate how the racemic mixture of beraprost and its isomers (−314d and −315d) can provide a protective, anti‐apoptotic effect on FECs. Additionally, this study aimed to determine the minimum effective dose of beraprost required to induce protective (anti‐apoptotic) effects in FECs.

## 2 Materials and Methods

### 2.1 Chemicals and Reagents

**FIGURE 1:**
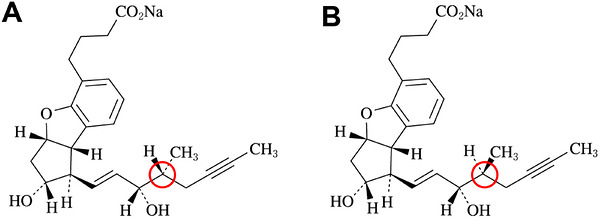
The chemical structures of the racemic beraprost, beraprost‐314d, and beraprost‐315d. Beraprost‐314d and its enantiomer (A) and beraprost‐315d and its enantiomer (B) are shown. The difference between the two stereoisomers is indicated by a red circle.

Pharmaceutical‐grade beraprost and separated beraprost‐314d and beraprost‐315d isomers were manufactured and provided by Toray Industries, Inc. (Tokyo, Japan). These chemical structures are shown in Figure 1. These were dissolved with UltraPure distilled water (Thermo Fisher Scientific, Waltham, MA, USA) at a concentration of 10 mM and sterilized with a 0.22‐µm filter. The solution was further diluted 10 times with the distilled water to 1 mM and kept at 4°C until use. Although chiral HPLC analysis was not performed in the present study to directly confirm post‐dissolution purity, the stereochemical stability of these isomers under these specific conditions has been validated by established protocols from previous pharmacological investigations (Shen et al. 2019). Because the compounds were maintained in a neutral aqueous solution at 4°C, the acidic conditions known to induce epimerization of prostaglandins were avoided. Furthermore, material data sheets provided by commercial suppliers confirm that these compounds remain stable in aqueous solutions for several weeks under refrigeration, ensuring their suitability and stability for our in vitro cell culture experiments.

### 2.2 Cell Line and Cell Culture Conditions

The FECs were purchased from Applied Biological Materials (Richmond, Canada). These were derived from healthy feline aortas and immortalized as previously described (Olyslaegers et al. 2013). Following the manufacturer's instruction, the cells were cultured in Prigrow III Medium containing 10% fetal bovine serum (FBS), 50 µg/mL endothelial cell growth supplement (ECGS), 0.1 mg/mL gentamycin sulphate, 1 mM sodium pyruvate, 1% (v/v) non‐essential amino acids, 10 U/mL heparin sodium, and 1% penicillin‐streptomycin mixture (“complete medium”). The complete medium was stored at 4°C and was warmed to room temperature (22°C) before use.

FECs were grown in 25‐cm^2^ culture flasks (Corning Inc., Corning, NY, USA) at 37°C in a humidified 5% CO_2_ incubator (Thermo Fisher Scientific) until sub‐confluency in the logarithmic growth phase was reached. After aspirating the medium, the cells were washed twice with Dulbecco's phosphate‐buffered saline (D‐PBS; 5 mL), detached with 0.25% (w/v) trypsin‐1 mM ethylenediaminetetraacetic acid (0.3 mL), and resuspended in complete medium (3 mL) to stop trypsinization. Cell suspensions were collected, and the cells were counted and split 1:3 to 1:4 in 25‐cm^2^ culture flasks for passage. After 3 days of culture, the cells at passage 3 (P3;population doubling level [PDL] 5.2) were cryopreserved in Cellbanker (Takara Bio Inc., Shiga, Japan) at –80°C until use. For further analysis, the frozen stored cells were thawed and cultured for 4 days. Subsequently, the cells were passaged and seeded in complete medium in 24‐well culture plates (TPP, Trasadingen, Switzerland) at a density of 4 × 10^5^ cells per well at P5, PDL10.1 per well (0.5 mL/well).

### 2.3 Determination of Caspase 3/7 Activity in FECs Under Various Starvation Culture Conditions

To establish the optimum culture condition for starvation‐induced apoptosis, six starvation media were prepared with varying concentrations of FBS (0.5%, 0.1%, or 0%), each with or without 50 µg/mL ECGS. FECs, which had been cultured for 43–44 h in complete medium, were washed once with D‐PBS (0.5 mL), and then either complete medium or one of six starvation media were added to the FECs in a 24‐well plate (triplicates per condition, 0.5 mL), and incubated for 4 h, before measuring the caspase‐3/7 activity, a key indicator of apoptosis (Taylor et al. 2008).

After 4 h of incubation, the medium was removed and replaced by ice‐chilled cell lysis buffer containing 20 mM 4‐(2‐hydroxyethyl)‐1‐piperazineethanesulfonic acid, 250 mM sucrose, 50 mM potassium chloride, 2.5 mM magnesium chloride, and 1 mM dithiothreitol (0.2 mL/well). The plates were then frozen at –80°C followed by thawing at room temperature (22°C) once. The cell lysates were sonicated on ice using an ultrasonic disruptor UR‐21P (Tomy, Tokyo, Japan) at a minimum power level (0–1) for 10‐ to 20‐s pulse. Caspase‐3/7 activity was measured in 96‐well plates (Corning) using the Apo‐ONE Homogeneous Caspase‐3/7 Assay (Promega, Madison, WI, USA) according to the manufacturer's protocol. The fluorescence intensities at 485 and 530 nm were obtained using a SpectraMax M3 plate reader (Molecular Devices, San Jose, CA, USA), and the results were normalized to the protein content quantified using the Bradford assay (Bio‐Rad, Hercules, CA, USA). The complete medium group served as a control (relative caspase‐3/7 activity, % of complete‐medium control).

### 2.4 Determination of Caspase‐3/7 Activity in Starved FECs and Upon Treatment With Beraprost

FECs were cultured as described above and transferred to 24‐well plates. The cells were incubated for 4 h with either 0.5 mL of complete medium (without beraprost), starvation medium (0.1% FBS without ECGS) without beraprost, or starvation medium with one of four different concentrations of beraprost, beraprost‐314d, or beraprost‐315d, which were selected based on previous studies (Kajikawa et al. 1989; Kainoh et al. 1992). Each condition was added to a total of nine wells, resulting in three independent triplicate experiments. After incubation, the caspase‐3/7 activity was measured as described above.

### 2.5 Quantification of *Bax* and *Bcl2* Expression in Starved FECs and Upon Treatment With Beraprost

The cells were prepared for RNA extraction in separate six‐well culture plates. Prior to this, FECs were incubated for 4 h with either 2.0 mL of complete medium, starvation medium, or starvation medium with one of four concentrations (0.1, 0.5, 1.0, or 5.0 nM) of beraprost (racemic mixture). After incubation, the medium was carefully removed and replaced by 350 µL of the lysis buffer provided by NucleoSpin RNA (Macherey‐Nagel, Düren, Germany). RNA was extracted and purified according to the manufacturer's instructions. The extracted RNA was reverse‐transcribed using the ReverTra AceTM qPCR RT Master Mix (Toyobo Co., Ltd., Osaka, Japan). The complementary DNA (cDNA) was stored at –20°C until use.

Relative gene expression was quantified using THUNDERBIRD Next SYBRTM qPCR Mix (Toyobo Co., Ltd.) on an Applied Biosystems 7500 Real‐Time PCR System (Thermo Fisher Scientific). The oligonucleotide primers used for the amplification of the target genes were as follows: β‐actin (Jursza et al. 2014) (F: ATCAAGGAGAAGCTGTGCTACGT; R: CGTTGCCGATGGTGATCA), *Bax* (Zhao et al. 2008) (F: CCGATGGCAACTTCAACTGGG; R: GTCAGCACTCCCGCCACAAAG), and *Bcl2* (Zhao et al. 2008) (F: GGGATGGCGTGAACTGG; R: CAGGAGAAATCAAACAGAGGC). All assays were performed three times, and the relative expression of mRNA was calculated using the 2‐ΔΔCt method. β‐actin was used as the reference gene for mRNA.

### 2.6 In Situ Cell Death Detection via Terminal Deoxynucleotidyl Transferase‐Mediated dUTP Nick End Labelling

For transferase dUTP nick end labelling (TUNEL) staining, FECs were seeded onto 4‐well culture slides (Corning) until they reached confluence. Following the same starvation/treatment protocol as in the gene expression assay described above, the cells were incubated for 4 h before replacing the culture medium with ice‐cold 4% paraformaldehyde (Fujifilm Wako Pure Chemical Corp., Osaka, Japan) for cell fixation. The slides were incubated at 4°C for 25 min and were subsequently permeabilized using 0.2% Triton X‐100 (Sigma‐Aldrich, St. Lois, MO, USA) for 5 min at room temperature. TUNEL staining was performed routinely using the DeadEnd Fluorometric TUNEL System (Promega), as recommended by the manufacturer. The stained culture slides were then covered with a clean cover glass (24 × 24 mm; Matsunami, Osaka, Japan) using DAPI Fluoromount‐G mounting medium (Southern Biotechnology Associates, Birmingham, AL, USA) to counterstain the cell nuclei. The number of apoptotic cells was determined by counting the number of green fluorescent cells following a modified method of Akbar and Kim (2002). Briefly, at least 150 cells were counted in four random fields per replicate using a Keyence All‐in‐One Fluorescence microscope BZ‐X810 (Keyence Ltd., Osaka, Japan). The percentage of TUNEL‐positive cells was determined based on the total number of cells counted per replicate.

### 2.7 Statistical Analysis

All experiments were done in biological triplicate. Each consisted of three technical replicates, which were averaged prior to statistical analysis. One‐way analysis of variance was used to compare the means of multiple groups. The differences between starvation conditions were tested by a two‐sided Dunnett's test to compare each treatment concentration with the starvation control. The differences in effects among the three treatment groups were tested by a two‐tailed Holm–Bonferroni test. The significance levels were set at *p* < 0.05.

## 3 Results

### 3.1 Establishment of Starvation Conditions in FECs to Induce Apoptosis‐Associated Events

To establish the appropriate culture medium conditions for activating caspase‐3/7, FECs were incubated with different amounts of FBS with or without ECGS supplementation. The complete medium group served as a control (relative caspase‐3/7 activity, % of complete‐medium control). Mean caspase‐3/7 activity in the starvation group ranged from 134.2% to 215.1% (Table S1). Although formal statistical analysis was omitted due to the pilot nature and limited power of this evaluation, a clear trend of caspase‐3/7 activation was observed in FECs following the reduction or removal of FBS/ECGS supplementation (Figure S1).

Based on these results, 0.1% FBS without ECGS was selected as the most suitable starvation condition for the subsequent pharmacological experiments. This condition containing 0.1% serum (FBS) was chosen to address the risk of disrupting endogenous prostacyclin function, which can occur under completely serum‐free conditions, as has been reported in human endothelial cells (Spector et al. 1985).

### 3.2 Inhibitory Effects of Beraprost, and Its Component Isomers Beraprost‐314d, and Beraprost‐315d on Caspase‐3/7 Activity in FECs

**FIGURE 2:**
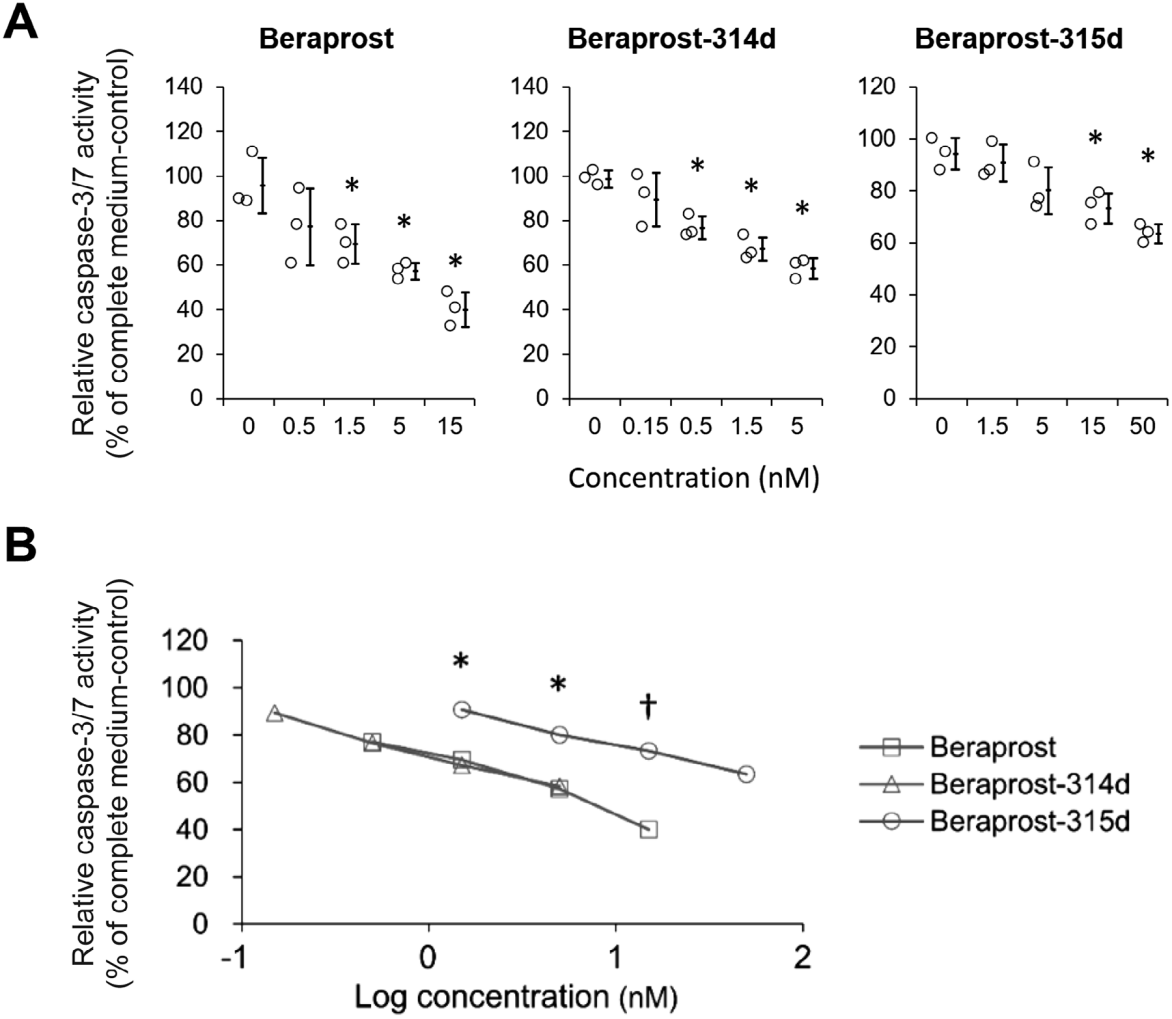
Effects of beraprost on starvation‐induced caspase‐3/7 activation in FECs. (A) The data represent dot plots and means ± SD (n = 3). *p < 0.05 vs. control by a two‐sided Dunnett's test. Control is the difference in caspase‐3/7 activity between complete medium and starvation medium (0.1% FBS alone). (B) a combined view of the graphs of beraprost, beraprost‐314d, and beraprost‐315d. ^*^ *p* < 0.001 among three groups by two‐tailed Holm–Bonferroni‐corrected *t‐*test. ^†^ *p* < 0.001 between two groups by two‐tailed Student's *t*‐test. FBS, fetal bovine serum; FEC, feline endothelial cell; SD, standard deviation.

Starvation‐induced activation of caspase‐3/7 was significantly inhibited by treatment with beraprost or beraprost‐314d at concentrations of 0.5 nM or higher. In the beraprost‐315d group, inhibitory effects were observed at a concentration of 5 nM (*p* < 0.025) (Figure 2A). Caspase 3/7 activity levels in the beraprost group were nearly identical to those in the beraprost‐314d group (Figure 2B), whereas activity levels were significantly higher in the beraprost‐315d group. Additional details are provided in Table S2.

### 3.3 *Bax/Bcl2* Expression in Starved FECs When Treated With Beraprost

**FIGURE 3:**
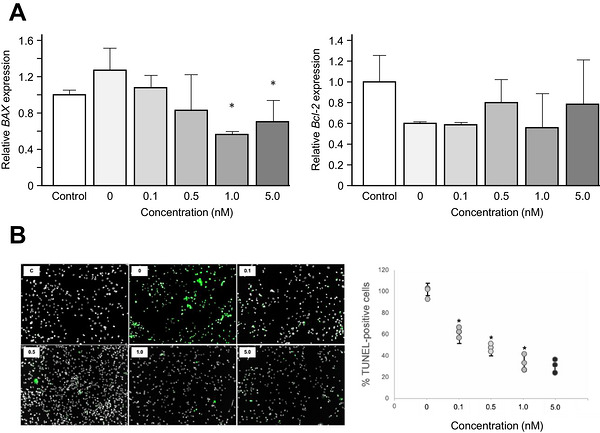
Effects of beraprost against apoptosis‐associated events in FECs. (A) Relative expression of *Bax* and *Bcl2*, apoptosis‐related genes in serum‐starved FECs. The data represent the mean ± SD. ^*^ *p* < 0.05 vs. control by a two‐sided Dunnett's test. (B) Detection of apoptotic cell numbers using TUNEL staining assay. Representative fluorescence images of TUNEL‐stained (green) FECs under different concentrations of beraprost during serum starvation are shown. The data represent dot plots and mean ± SD. All data are expressed as % of the control. ^*^ *p* < 0.001 vs. beraprost 0 nM by two‐sided Dunnett's test. C represents non‐starved control cells. FEC, feline endothelial cell; SD, standard deviation; TUNEL, terminal deoxynucleotidyl transferase dUTP nick end labeling.

The mean relative gene expression of pro‐apoptotic *Bax* in FECs under starvation showed an inverse relationship with the beraprost concentration (Figure 3A). Significant downregulation of *Bax* expression in starved FECs was apparent after beraprost treatment at 1.0 nM or higher (*p* < 0.05) compared to the non‐treated group. Treatment with beraprost or its isomers, even at high concentrations, did not cause significant changes in the expression of the anti‐apoptotic *Bcl2*.

### 3.4 DNA Fragmentation as Indicated by TUNEL Assay in Starved FECs When Treated With Beraprost

Apoptosis‐associated DNA fragmentation was confirmed in starved FECs without beraprost treatment, based on the percentage of TUNEL‐positive cell nuclei (Figure 3B). Treatment of starved FECs with beraprost significantly reduced the number of TUNEL‐positive apoptotic cells compared to starved FECs without beraprost treatment. At concentrations as low as 0.1 nM, the number of TUNEL‐positive cells was significantly reduced (*p* < 0.01); however, no further reduction of TUNEL‐positive cells was achieved at beraprost concentrations above 1.0 nM.

## 4 Discussion

The purpose of this study was to establish an in vitro model to evaluate the pharmacological effects of beraprost and its stereoisomers on FECs under starvation conditions and to use this model to assess the efficacy and mode of the anti‐apoptotic effect of beraprost in these cells. The optimal starvation conditions for FECs under which apoptosis‐associated events occurs were identified by measuring caspase 3/7 activity in FECs under various nutrient‐deprived cell culture conditions. When investigating the pharmacological anti‐apoptotic effects of beraprost against starvation‐induced cell death of FECs, the racemic mixture of beraprost and its isomer, beraprost‐314d, exhibited better caspase inhibitory efficacy than beraprost isomer 315d. The anti‐apoptotic effect of beraprost in this FEC model was confirmed using TUNEL staining assay. Furthermore, quantitative analysis of the relative *Bax* expression revealed a dose‐dependent downregulation with beraprost treatment.

Immortalized cell lines often have higher survival and resistance to apoptosis than freshly isolated cells, and successful attempts have been made to induce apoptosis in immortalized endothelium‐derived cell lines such as human EA.hy926 cells (Jia et al. 2012). However, endothelial cells display different biological behaviours depending on their origin (Thorin and Shreeve 1998). Whether FEC lines created by a relatively robust immortalization protocol via ectopic expression of the SV40LT antigen and hTERT can be used to induce apoptosis under these culture conditions has not been explored. ECGS and FBS contain nutrients such as proteins and growth factors necessary for the maintenance and proliferation of FECs in vitro. The present findings demonstrate that deprivation or reduction of ECGS and FBS activates caspase‐3/7 and induces apoptosis‐associated events in FECs. Although starvation using 0.1% FBS alone was selected as the most suitable condition for the pharmacological study of beraprost, all the presented conditions can be applied to various future pharmacological studies on feline endothelial functions.

Beraprost is a racemate of four enantiomers, and the individual isomers have been shown to represent different affinities and pharmacodynamic properties (Kajikawa et al. 1989; Shen et al. 2019). The previous comparative studies between the pharmacodynamics of beraprost‐314d and beraprost demonstrate the following: (1) the estimated half maximal effective concentration (EC50) for relaxation involving endothelial‐derived NO production in rat pulmonary artery was similar between the groups (beraprost 4.6 µM vs. beraprost‐314d 4.7 µM) and (2) EC50 of elevation of cAMP in human pulmonary arterial smooth muscle cells was 4.6‐fold lower in beraprost‐314d compared to beraprost. Therefore, isomer separation of beraprost to yield beraprost‐314d has been proposed to greatly affect its pharmacology (Shen et al. 2019). However, in human medicine, a randomized, double‐blind, placebo‐controlled phase 3 trial of oral administration of beraprost‐314d combined with inhaled treprostinil was carried out to examine the time to clinical worsening from baseline, but the outcome failed to achieve statistical significance (Oudiz et al. 2019). In the present study, the inhibitory effects of beraprost‐314d on starvation‐induced activation of caspase‐3/7 in FECs were almost consistent with those of beraprost, which correspond to previously described endothelial‐derived NO production in rat pulmonary arteries (Shen et al. 2019). The pharmacological target of beraprost in feline CKD has been assumed to be mainly the endothelium rather than the vascular smooth muscle based on a rodent study (Goto et al. 2014), which is supported by evidence that the clinical dose of beraprost does not show changes in hemodynamic parameters in healthy cats (Takenaka et al. 2018). Consequently, while further in vivo validation is required, our present results lead to the hypothesis that enantiomer isolation may not necessarily provide a substantial advantage in improving the clinical effectiveness of beraprost in patients with feline CKD.

However, the specific mechanism by which beraprost induces its anti‐apoptotic effects was not investigated in this study. Beraprost is involved in endothelial cell maintenance against oxidative stress in vitro (Kainoh et al. 1992) and in vivo in CKD and diabetes rodent models (Goto et al. 2014; Matsumoto et al. 2002). Beraprost has also been shown to stimulate prostacyclin signalling and nitric oxide (NO) production through protein kinase A and cyclic adenosine monophosphate (cAMP) signalling in human and bovine endothelial cells (Niwano et al. 2003). Prostacyclin is released from the endothelium, bound to cellular and nuclear receptors of endothelial cells, and regulates cell survival as an autocrine factor (Narumiya et al. 1999 Lim and Dey 2002; Cines et al. 1998; Hoffmann et al. 2001). CKD is commonly diagnosed in aged cats (Sparkes et al. 2016). Aging of endothelial cells leads to enhanced induction of apoptosis due to reduced NO synthesis, which is proposed to cause further endothelial; cell aging and result in a viscous cycle (Hoffmann et al. 2001). Given the above and the present findings, we hypothesize that the attenuation of apoptosis‐associated events by beraprost may represent a potential mechanism for preserving the capillary network in feline CKD kidneys. However, whether this involves the regulation of the Bax and caspase‐3 pathways via NO or prostacyclin signalling in vivo remains elusive and requires further investigation.

There are several limitations of this study. The in vitro starvation model used in this study does not fully recapitulate the complex microenvironment of feline CKD kidneys, which involves uremic toxins, hemodynamic alterations, and intricate cell–cell interactions. While multiple complementary approaches (caspase‐3/7 activity, TUNEL staining, and Bax expression) were used to evaluate cell death, these assays have limitations and may not definitively rule out other forms of cell death or DNA damage caused by severe starvation stress. While our data clearly demonstrate that beraprost attenuates these apoptosis‐associated events in cultured FECs, it remains to be determined whether this suppression directly contributes to the prevention of capillary rarefaction or the slowing of kidney dysfunction in cats with CKD. Our current findings should be considered preliminary. Further investigations, including in vivo studies and experiments utilizing primary feline renal endothelial cells, are necessary to validate the direct clinical relevance of these findings to feline CKD.

Taken together, these findings indicate that beraprost treatment inhibits apoptosis‐associated events in cultured FECs, even at low concentrations. These findings contribute to the understanding of the mode of action of beraprost in terms of its anti‐apoptotic and endothelial‐protective effects, which may be relevant to cats with CKD. In the future, to further explore the clinical relevance of this study, it will be necessary to confirm the current findings in feline patients with CKD using clinicopathological markers associated with endothelial injury and NO dysregulation, as proposed in humans (Jourde‐Chiche et al. 2019), and pathological biopsies.

## 5 Conclusions

Nutrient deprivation in cell culture media induces caspase‐3/7 activity and increases apoptosis‐associated events in FECs. Beraprost dose‐dependently inhibited caspase‐3/7 activation. The minimum effective dose of the isomer beraprost‐314d was consistent with that of beraprost, whereas that of the isomer beraprost‐315d was higher. Beraprost inhibited caspase 3/7 activity even at low concentrations, downregulated *Bax* expression, and inhibited FEC death.

## Author Contributions

T.M. contributed to data curation, formal analysis, investigation, methodology, validation, visualization, and writing – original draft. C.N.B.B.M. contributed to data curation, formal analysis, investigation, visualization, and writing – original draft. T.Y. contributed to conceptualization, data curation, formal analysis, funding acquisition, supervision, validation, visualization, and writing – review and editing.

## Funding

The authors have nothing to report.

## Conflicts of Interest

T.M. is an employee of Toray Industries, Inc. The remaining authors declare no conflicts of interest.

## Ethics Statement

Since this study was conducted exclusively using an established feline cell line and did not involve any procedures utilizing live animals or primary tissue collections, ethical approval was not required.

## Supporting information




**Supporting File 1**: vms371127‐sup‐0001‐tableS1‐S2.docx

**Supporting File 2**: vms371127‐sup‐0002‐figureS1.docx

## Data Availability

Additional data are available from the corresponding author upon reasonable request.
