# Supplementary material for: Beraprost Attenuates Starvation‐Induced Apoptosis and Protects Feline Endothelial Cells
Source: Vet Med Sci. 2026 Aug 10;12(5):e71127. doi: 10.1002/vms3.71127 (PMC13455532; doi:10.1002/vms3.71127)
Supplement: Supplementary file 1 [file VMS3-12-e71127-s001.docx]

**Supplemental Table 1.** Different culture conditions for FECs and caspase activity

| **Group** | **FBS** | **ECGS**  **[50 µg/mL]** | **Mean caspase-3/7 activity***  **[Relative % of complete medium]**** | |
| --- | --- | --- | --- | --- |
| Complete medium | 10% | + | 100 | |
| Starvation medium | 0.5% | + | 134.2 | |
|  | 0.5% | - | 143.9 | |
|  | 0.1% | + | 159.0 | |
|  | 0.1% | - | 188.9 | |
|  | 0% | + | 164.0 | |
|  | 0% | - | 215.1 |  |
| *Mean of triplicate measurements  **the complete medium group was used as 100% control; starvation groups were referred to as % of control. | | | | |

**Supplemental Table 2.** Caspase-3/7 activity in IFECLs treated with beraprost

| **Group** | | **Mean caspase-3/7 activity***  **[% of complete medium-control]**** | |
| --- | --- | --- | --- |
| Beraprost | 0 nM | 99.3 ± 5.8 | |
|  | 0.5 nM | 85.8 ± 4.9 | |
|  | 1.5 nM | 74.0 ± 3.3 | |
|  | 5 nM | 59.2 ± 3.8 | |
|  | 15 nM | 44.1 ± 3.4 |  |
| Beraprost-314d | 0 nM | 98.7 ± 3.8 | |
|  | 0.15 nM | 89.5 ± 4.5 | |
|  | 0.5 nM | 76.6 ± 2.9 | |
|  | 1.5 nM | 67.2 ± 2.5 | |
|  | 5 nM | 58.3 ± 3.6 |  |
| Beraprost-315d | 0 nM | 94.2 ± 2.2 | |
|  | 1.5 nM | 90.8 ± 2.9 | |
|  | 5 nM | 80.1 ± 4.1 | |
|  | 15 nM | 73.3 ± 3.2 | |
|  | 50 nM | 63.4 ± 2.3 |  |
| *Mean of n = 3, three independent triplicate experiments ± standard deviation (SD)  **the starvation medium (0.1% FBS alone) group was used as 100% control; treatment groups were referred to as % of control. | | | |
