# Supplementary material for: Beraprost Attenuates Starvation‐Induced Apoptosis and Protects Feline Endothelial Cells
Source: Vet Med Sci. 2026 Aug 10;12(5):e71127. doi: 10.1002/vms3.71127 (PMC13455532; doi:10.1002/vms3.71127)
Supplement: Supplementary file 2 [file VMS3-12-e71127-s002.docx]

**Supplemental Fig. S1.** Comparison of mean group differences of caspase 3/7 activity. Although formal statistical analysis was omitted due to the pilot nature and limited power of this evaluation, a clear trend of caspase-3/7 activation was observed in FECs following the reduction or removal of FBS/ECGS supplementation.
